# Digital Isolation and Depression Risk in Older Adults Using the National Health and Aging Trends Study Database: 8-Year Longitudinal Study

**DOI:** 10.2196/75174

**Published:** 2025-12-12

**Authors:** Zhili He, Shijun Yang, Wei Tong, Wei Zhang

**Affiliations:** 1 Department of Neurology, The Central Hospital of Wuhan, Tongji Medical College, Huazhong University of Science and Technology, 26 Shengli Street, Jiang'an District, Wuhan, 430014, China, 86 02765699875; 2 Department of Cardiology, Union Hospital, Tongji Medical College, Huazhong University of Science and Technology, Wuhan, China; 3 Department of Orthopaedics, Union Hospital, Tongji Medical College, Huazhong University of Science and Technology, Wuhan, China

**Keywords:** digital isolation, depression, older adults, longitudinal cohort study, National Health and Aging Trends Study, NHATS, mental health, social isolation, technology use, risk factors, public health interventions

## Abstract

**Background:**

The rapid advancement of digital technologies has profoundly transformed communication practices. However, this technological revolution has also led to “digital isolation,” a form of social disconnection caused by limited or absent engagement with digital communication tools, including smartphones, computers, email, and the internet. This issue is particularly concerning for older adults, as it may increase their likelihood of developing mental health disorders, with depression being a primary concern. Although digital isolation has been studied less frequently than traditional social isolation, it may be a significant contributor to both the initiation and progression of depression in this population.

**Objective:**

This investigation seeks to assess longitudinal relationships between multidimensional digital disengagement (encompassing 4 dimensions: mobile device use, computer interaction, electronic correspondence, and web-based engagement) and incident depression among older adults, using longitudinal data from the nationally representative National Health and Aging Trends Study (NHATS).

**Methods:**

The analysis was conducted based on the NHATS dataset, a nationally representative longitudinal survey using multistage sampling to represent community-dwelling Medicare beneficiaries aged 65 years and older in the United States. We analyzed data from 2011 (Round 1) to 2018 (Round 8), including 8199 participants in the discovery and validation cohorts. Digital isolation was measured using a 4-item index based on self-reported nonuse of mobile phones, computers, email, and the internet. Participants were categorized into high (aggregate score ≥3) or low (aggregate score ≤2) digital isolation groups. Weighted Cox regression models with proportional hazards assumptions were used to quantify longitudinal associations between the digital isolation index (and its individual components) and incident depression, incorporating multivariable adjustment for sociodemographic characteristics (age, sex, and race or ethnicity), socioeconomic indicators (education level, family income, and marital status), and clinical profiles (tobacco use history and multimorbidity burden). Time-to-event analyses were visualized through Kaplan-Meier estimators, complemented by prespecified subgroup analyses evaluating effect modification patterns through interaction term testing.

**Results:**

A high level of digital isolation, as measured by the composite index, was associated with a significantly greater risk of incident depression (fully adjusted model: hazard ratio 1.35, 95% CI 1.18‐1.55; *P*<.001). Furthermore, analysis of the individual components showed that nonuse of computers, email, and the internet was each significantly associated with a higher depression risk, whereas mobile phone isolation had a weaker, nonsignificant association.

**Conclusions:**

The study revealed a robust association between increased digital isolation and a higher likelihood of depression in the older population. These results underscore the importance of implementing tailored public health strategies to address digital isolation, especially for older adults. To minimize its detrimental effects on mental health, policymakers should encourage digital literacy programs and strengthen mental health services.

## Introduction

In today’s digital era, the rapid advancement of information technology has profoundly reshaped human lifestyles. The increasing reliance on digital technologies for communication and social connection has led to the emergence of a phenomenon known as “digital isolation” [1]. It is important to distinguish the concept of “digital isolation” from the more widely recognized “digital divide” [2]. The digital divide typically refers to disparities in access to information and communication technologies and the necessary digital skills across populations [3]. In this study, we use the term “digital isolation” to describe a related but conceptually distinct psychosocial phenomenon: a state of social disconnection resulting from limited or absent engagement with essential digital communication tools, regardless of access [4]. This term refers to social disconnection that occurs when individuals either lack access to or refrain from using modern communication tools, such as smartphones, computers, email, and the internet. Although designed to foster human interaction, these technologies can paradoxically exacerbate loneliness and social isolation when misused or unavailable [4,5]. This paradox is of particular concern due to its potential negative impact on mental health, especially depression.

Depression, a common global mental health disorder, substantially impairs quality of life and social functioning. According to the World Health Organization, depression affects approximately 280 million individuals, or 3.8% of the global population, making it a significant public health concern [6]. Women are disproportionately affected compared to men, and prevalence increases significantly among older adults, where it is a leading cause of disability and suicide [7,8]. Beyond its psychological dimensions, depression is closely linked to physical health conditions such as cardiovascular disease and diabetes, contributing to increased mortality [9,10]. Therefore, identifying risk factors for depression and developing effective prevention and treatment strategies are crucial for public health.

The urgency of this issue is further underscored by the broader public health crisis of social isolation among older adults. A landmark 2020 report by the National Academies of Sciences, Engineering, and Medicine identified social isolation and loneliness as serious yet underrecognized public health risks, noting that approximately one-quarter of community-dwelling Americans aged 65 years and older are socially isolated [11]. The report emphasizes that social isolation poses a major risk for premature mortality, comparable to well-known risk factors such as smoking or obesity [12].

In the era of rapid digitalization, the long-standing problem of social isolation is acquiring a new and critical dimension. As social networks, health care resources, and daily services increasingly move online, limited digital engagement may become a key driver of social exclusion. Consequently, digital isolation—a form of social disconnection arising from nonuse of digital tools—may serve as a crucial pathway through which modern societal structures adversely affect mental health in vulnerable older adults [13,14]. While overuse of digital media poses risks for some, older adults are more often affected by digital underuse or exclusion, which may compound traditional social isolation and increase the risk of depression [15]. Research has begun to explore how digital isolation contributes to depressive symptoms among older adults, but evidence remains limited, particularly regarding the combined effects of multiple forms of digital isolation and their cumulative impact on mental health [16,17].

We conceptualize digital isolation as a latent psychosocial state; to make it observable, we use a 4-domain nonuse composite (mobile phone, computer, email, and internet) as an observable proxy, distinct from the digital divide and digital literacy. Within this framework, this study aims to assess the longitudinal association between multidimensional digital isolation—defined by the use or nonuse of mobile phones, computers, email, and the internet—and the risk of incident depression in a large, nationally representative cohort of older adults. Using data from a large-scale retrospective cohort, we aim to examine both the individual and combined effects of these types of digital disengagement on depression risk. The findings are expected to yield new insights into the mental health consequences of digital disconnection and support the development of targeted public health interventions.

## Methods

### Study Population

The National Health and Aging Trends Study (NHATS) formed our analytical sample, a longitudinal survey that uses a multistage probability sampling technique to mirror the US population of community-living Medicare beneficiaries aged 65 years and older [18].

We analyzed data collected from NHATS starting in 2011 (Round 1). For the discovery cohort, we used data from Round 1 (2011) to Round 8 (2018), encompassing 7 waves of follow-up. The initial wave enrolled 8245 participants. To ensure analytical precision, we excluded 2230 participants lacking crucial information on key variables such as digital isolation and depressive symptoms, which were central to our research question. Additionally, we excluded 846 participants with a baseline diagnosis of depression to focus on the de novo development of depression. This resulted in a discovery cohort of 5169 participants.

For external validation, we established an independent verification cohort comprising 4182 newly enrolled participants during Wave 5 (2015) of NHATS data collection, ensuring temporal and spatial independence from the discovery sample. For these participants, we used data from Round 5 to Round 12 (2015‐2022), also spanning 7 waves of follow-up. The validation cohort was formed by excluding 723 individuals who had missing data on crucial variables and an additional 429 participants who were diagnosed with depression at the baseline assessment. Consequently, a total of 3030 participants constituted the final validation cohort.

In the final analysis, we combined the discovery and validation cohorts, yielding a total sample of 8199 participants. Merging these cohorts enhanced the robustness and generalizability of our findings.

### Digital Isolation

Digital isolation was defined as limited engagement with modern communication technologies. Based on NHATS data, we assessed 4 domains of digital engagement: mobile phone ownership, computer use, email or text messaging, and internet activity. We consider multidomain nonuse as an observable proxy for the latent state of digital isolation, mirroring the common practice in social isolation research where concrete behaviors are used to represent abstract psychosocial states. This multidomain operationalization aligns with evidence framing older adults’ digital engagement as a continuum and supports using composite, multidomain indicators to capture disengagement [19]. Each domain was coded as 1 (nonuse or infrequent use) or 0 (regular use), yielding a cumulative score ranging from 0 to 4. The specific NHATS questionnaire items, variable names, and precise coding logic used to construct each of these 4 components are detailed in Table S4 (Multimedia Appendix 1). Higher scores reflected greater levels of digital isolation. Participants with scores ≥3 were classified as highly digitally isolated, in line with prior NHATS-based gerontechnology research that stratified older adults by multidomain technology nonuse [5]. This threshold approximately corresponded to the top quartile of the score distribution and indicated substantial disengagement from the digital environment.

### Depression

The NHATS used the Patient Health Questionnaire-2 (PHQ-2) to evaluate depressive symptoms. This concise screening instrument consists of 2 questions that assess the frequency of such symptoms experienced over the previous month: (1) a lack of interest or enjoyment in activities and (2) persistent feelings of sadness, hopelessness, or depression. Respondents can choose from a scale of “not at all” (assigned 1 point) to “nearly every day” (4 points), resulting in a total PHQ-2 score between 2 and 8. Higher scores on this scale indicate more severe depressive symptoms [20,21]. Notably, NHATS applies a 1-month reference period for the PHQ-2, differing from the standard 2-week timeframe, to align with the reference periods used for other functional measures, such as self-care and mobility assessments [22]. Based on the foundational work of Kroenke et al [20], we used a threshold of 3 points or more to define clinically significant depressive symptoms.

### Covariates

Our analytical approach comprehensively adjusted for potential confounders spanning sociodemographic determinants, socioeconomic gradients, behavioral risk profiles, and clinical comorbidities. The analysis focused on several variables known from previous research to be linked with depression and mental health. These variables comprised age, categorized as ≤75 years and >75 years; gender, distinguished as male and female; race or ethnicity, comparing non-Hispanic Whites with other groups; marital status, with comparisons made between married individuals and others; educational attainment, contrasted as less than high school versus high school or above; income level, separated into <50,000; smoking status, classified as nonsmokers and smokers; presence or absence of sleep disorders; dementia status; presence or absence of anxiety; and chronic disease burden, defined as having <3 or ≥3 conditions.

Age, gender, and race or ethnicity may affect individuals’ use and adaptation to digital technologies, influencing their social interaction patterns and mental well-being. Marital status, education level, and income level were included as proxies for socioeconomic status, a well-documented determinant of mental health outcomes [23,24]. Additionally, health behaviors and medical history, such as smoking status, sleep disorders, dementia, and anxiety, have shown strong associations with the risk of depression in the existing literature [25,26].

### Statistical Analysis

We used survey-weighted analytical methods to address the intricate design of the NHATS dataset. These approaches incorporated adjustments for varying selection probabilities, strategies to minimize nonresponse bias, and targeted oversampling of the specific population segments. Specifically, all analyses incorporated the cross-sectional analytic weights provided in the NHATS public use files to generate nationally representative estimates [18]. Weighted means with SE were used to describe continuous measures, while categorical variables were depicted as adjusted proportions accompanied by 95% CIs. Intergroup comparisons used the chi-square test. Survival analyses used inverse probability-weighted Cox proportional hazards models, with hierarchical adjustment for: (1) sociodemographic factors (age, sex, race or ethnicity, and education), (2) health behaviors (tobacco exposure and sleep dysfunction), and (3) clinical profiles (multimorbidity burden, dementia status, and anxiety comorbidity). To clarify the independent effects of digital isolation components (eg, phone, computer, email, and online isolation) on depression risk, additional analyses estimated hazard ratios (HRs) for each component.

Kaplan-Meier (KM) survival and stratified analyses were conducted after combining the discovery and validation cohorts. KM curves visualized depression incidence differences between low- and high-isolation groups, with log-rank tests evaluating their statistical significance. To examine the appropriateness of combining discovery and validation cohorts, we constructed a dummy variable to indicate cohort membership and tested its interaction with digital isolation in Cox regression models. Nonsignificant interaction terms suggested that the cohort did not modify the effect, thus supporting the pooled analysis. In stratified analyses, the low-isolation group served as the reference, and associations between digital isolation and depression incidence were examined across subgroups based on age (<75 vs ≥75 years), gender, race or ethnicity, smoking status, sleep disorders, marital status, and chronic disease burden (<3 vs ≥3). To examine potential nonlinear associations between digital isolation scores and depression risk, we used restricted cubic splines (RCS) in our analysis. Cox regression models with 3 knots were fitted to assess nonlinear trends in digital isolation scores (0‐4). Risk curves visualized the progressive impact of higher digital isolation scores on depression risk.

To evaluate the robustness of the observed associations, 2 sets of sensitivity analyses were performed. First, the digital isolation score was recategorized into 3 levels—no isolation (0 points), moderate isolation (1‐2 points), and high isolation (3‐4 points)—and Cox proportional hazards models were re-estimated in the discovery, validation, and pooled cohorts. Second, to address potential confounding by offline social disengagement, we constructed an In-person Social Isolation Score (range: 0‐4) based on 4 dimensions: absence of a confidant, lack of participation in social or religious activities, and living alone. This variable was included in fully adjusted models to assess the independent effect of digital isolation. All analyses were performed using R software (version 4.4.0; R Core Team), with 2-sided *P* values <.05 considered statistically significant.

### Ethical Considerations

This study used deidentified, publicly available data from the NHATS. The original NHATS study protocol was approved by the Johns Hopkins University Institutional Review Board, and all participants provided written informed consent at enrollment. For the present secondary data analysis, the research protocol was additionally reviewed and approved by the Institutional Review Board of The Central Hospital of Wuhan, which is affiliated with Tongji Medical College and Huazhong University of Science and Technology. Because the NHATS public-use dataset contains no identifiable private information, no additional consent was required for this analysis. All procedures adhered to institutional, federal, and journal guidelines for research involving human participants.

## Results

### Baseline Characteristics of Participants

**Figure 1.**
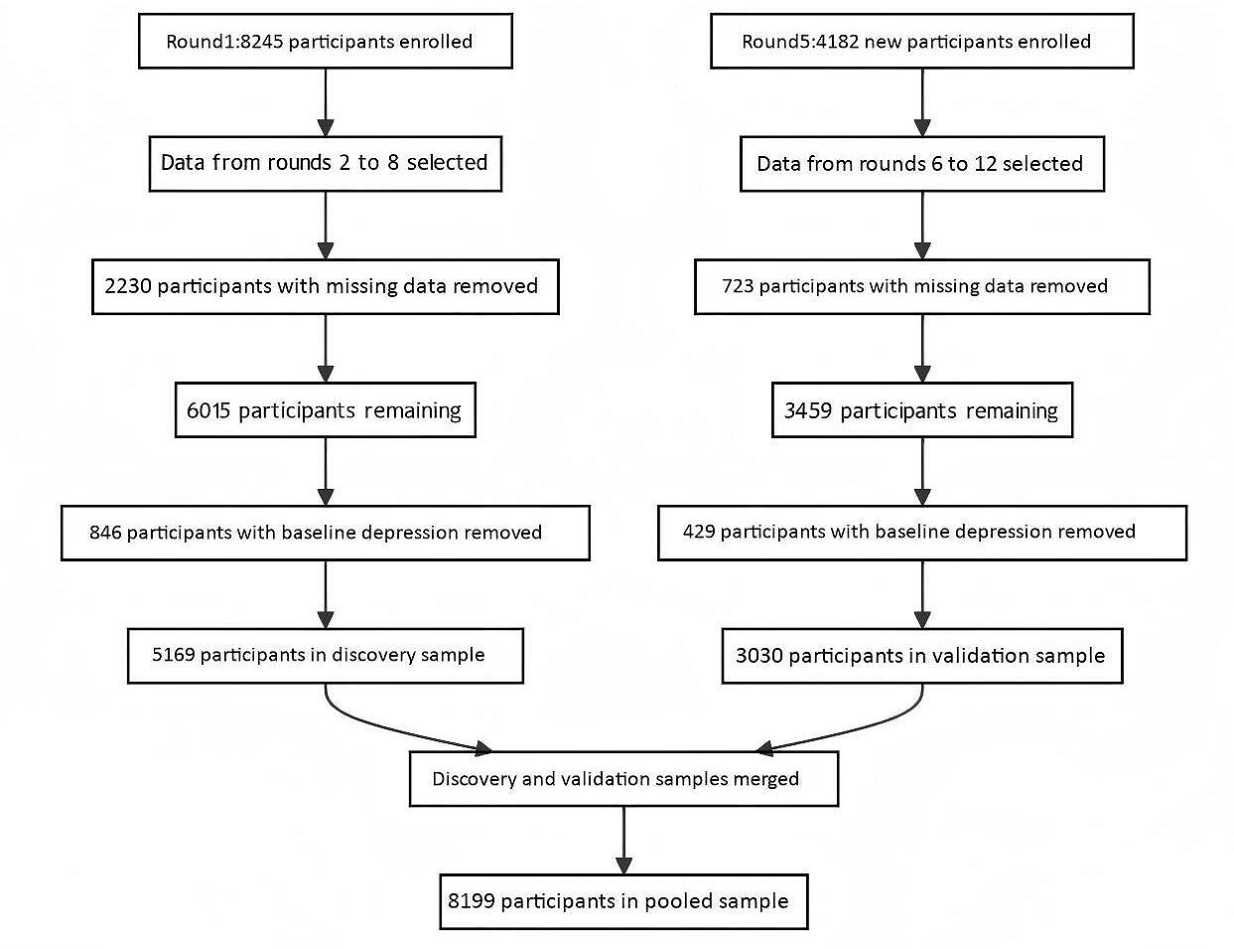
Flowchart of participant selection and data integration process for discovery, validation, and pooled samples.

As shown in Figure 1, a total of 8199 participants were included after applying the predefined inclusion and exclusion criteria. Table 1 presents the baseline sociodemographic and clinical profiles stratified by analytical cohort (discovery: n=5169, validation: n=3030, pooled: n=8199). The participant pool comprised 44.72% males and 55.28% females. In terms of age, the majority, 68.39%, were 75 years or younger, while the remaining 31.61% were older than 75 years. The majority (70.39%) identified as non-Hispanic White. Educational attainment indicated that 80.12% of participants had completed at least high school, while 17.89% had not. Income data revealed that 35.63% of participants had an annual income below US $50,000, whereas 21.20% reported an income above this threshold. Lifestyle analysis showed that 48.31% were nonsmokers, and 51.59% were smokers. Health assessments found that 80.89% of participants did not report sleep disorders, 97.46% were dementia-free, and 93.19% showed no symptoms of anxiety. Regarding chronic disease burden, 78.86% of participants had fewer than 3 chronic conditions, while 21.14% had 3 or more. For digital isolation, 69.01% of participants were categorized as low isolation and 30.99% as high isolation. Among specific components, 8.59% experienced phone isolation, 30.00% computer isolation, 52.14% email isolation, and 51.09% online isolation. Chi-square tests identified significant group differences in categorical variables, including gender, age group, education, income, smoking status, sleep disorders, dementia, anxiety symptoms, chronic disease burden, and components of digital isolation.

**Table 1.** Baseline characteristics of participants.^a^

Variables^b^	Discovery group (n=5169)	Validation group (n=3030)	Pooled group (n=8199)
Sex, n (%)			
Male	2303 (44.55)	1364 (45.02)	3667 (44.72)
Female	2866 (55.45)	1666 (54.98)	4532 (55.28)
Age group, n (%)^c^			
≤75 years	3440 (66.55)	2167 (71.52)	5607 (68.39)
>75 years	1729 (33.45)	863 (28.48)	2592 (31.61)
Race or ethnicity, n (%)^c^			
White, non-Hispanic	3699 (71.56)	2072 (68.38)	5771 (70.39)
Other^d^	1413 (27.34)	841 (27.76)	2254 (27.49)
Marital status, n (%)^c^			
Married	2836 (54.87)	1609 (53.1)	4445 (54.21)
Other	2327 (45.02)	1417 (46.77)	3744 (45.66)
Education, n (%)^c^			
Low (<high school)	997 (19.29)	470 (15.51)	1467 (17.89)
High (≥high school)	4118 (79.67)	2451 (80.89)	6569 (80.12)
Income, n (%)^c^			
Low (<50,000)	1919 (37.13)	1002 (33.07)	2921 (35.63)
High (≥50,000)	1019 (19.71)	719 (23.73)	1738 (21.2)
Smoking status, n (%)^c^			
Nonsmoker	2453 (47.46)	1508 (49.77)	3961 (48.31)
Smoker	2713 (52.49)	1517 (50.07)	4230 (51.59)
Sleep disorder, n (%)^c^			
No	4184 (80.94)	2448 (80.79)	6632 (80.89)
Yes	975 (18.86)	579 (19.11)	1554 (18.95)
Dementia, n (%)^e^			
No	5033 (97.37)	2958 (97.62)	7991 (97.46)
Yes	134 (2.59)	70 (2.31)	204 (2.49)
Anxiety, n (%)^c^			
No	4808 (93.02)	2833 (93.5)	7641 (93.19)
Yes	361 (6.98)	197 (6.5)	558 (6.81)
Chronic disease, n (%)^c^^,^^f^			
Less than 3 diseases	4060 (78.55)	2406 (79.41)	6466 (78.86)
3 or more diseases	1109 (21.45)	624 (20.59)	1733 (21.14)
Digital isolation group, n (%)^c^			
Low isolation	3408 (65.93)	2250 (74.26)	5658 (69.01)
High isolation	1761 (34.07)	780 (25.74)	2541 (30.99)
Items of digital isolation, n (%)			
Phone isolation^e^	533 (10.31)	171 (5.64)	704 (8.59)
Computer isolation^c^	1579 (30.55)	881 (29.08)	2460 (30)
Email isolation^c^	2973 (57.52)	1302 (42.97)	4275 (52.14)
Online isolation^c^	2856 (55.25)	1333 (43.99)	4189 (51.09)

a Percentages for some variables, such as Income and Marital Status, may not sum to 100% due to missing data. Participants with missing data on any key variable were excluded from the final analysis as described in the Methods.

b We conducted chi-square tests for all categorical variables to assess the association between each variable and the occurrence of depression.

c Variables with *P* values <.05 in chi-square tests are marked with superscript “c.”

d The “Other” category in the race or ethnicity classification includes Black or African American, American Indian, Alaska Native, Asian, Native Hawaiian, Pacific Islander, and other unspecified races or ethnicities. The “Other” category for Marital Status includes widowed, divorced, separated, and never married.

e Variables with *P* values <.001 in chi-square tests are marked with superscript “e.”

f The assessment of chronic disease burden is based on the cumulative number of the following conditions: heart disease, hypertension, arthritis, osteoporosis, diabetes, lung disease, stroke, dementia or Alzheimer disease, cancer, and others.

### Association Between Digital Isolation and Depression

**Figure 2.**
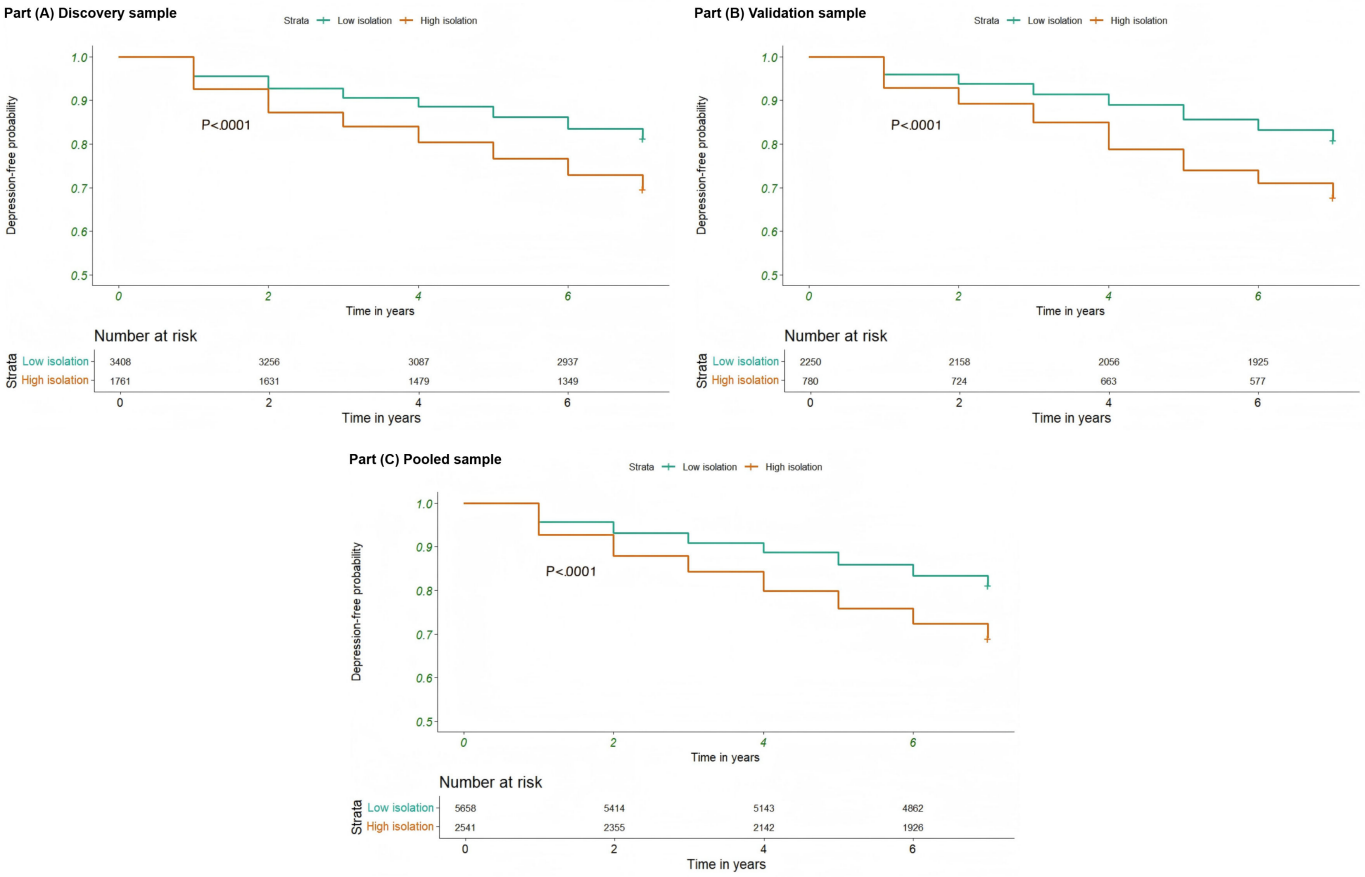
Kaplan-Meier curves depicting the identified association between digital isolation and the risk of depression.

**Figure 3.**
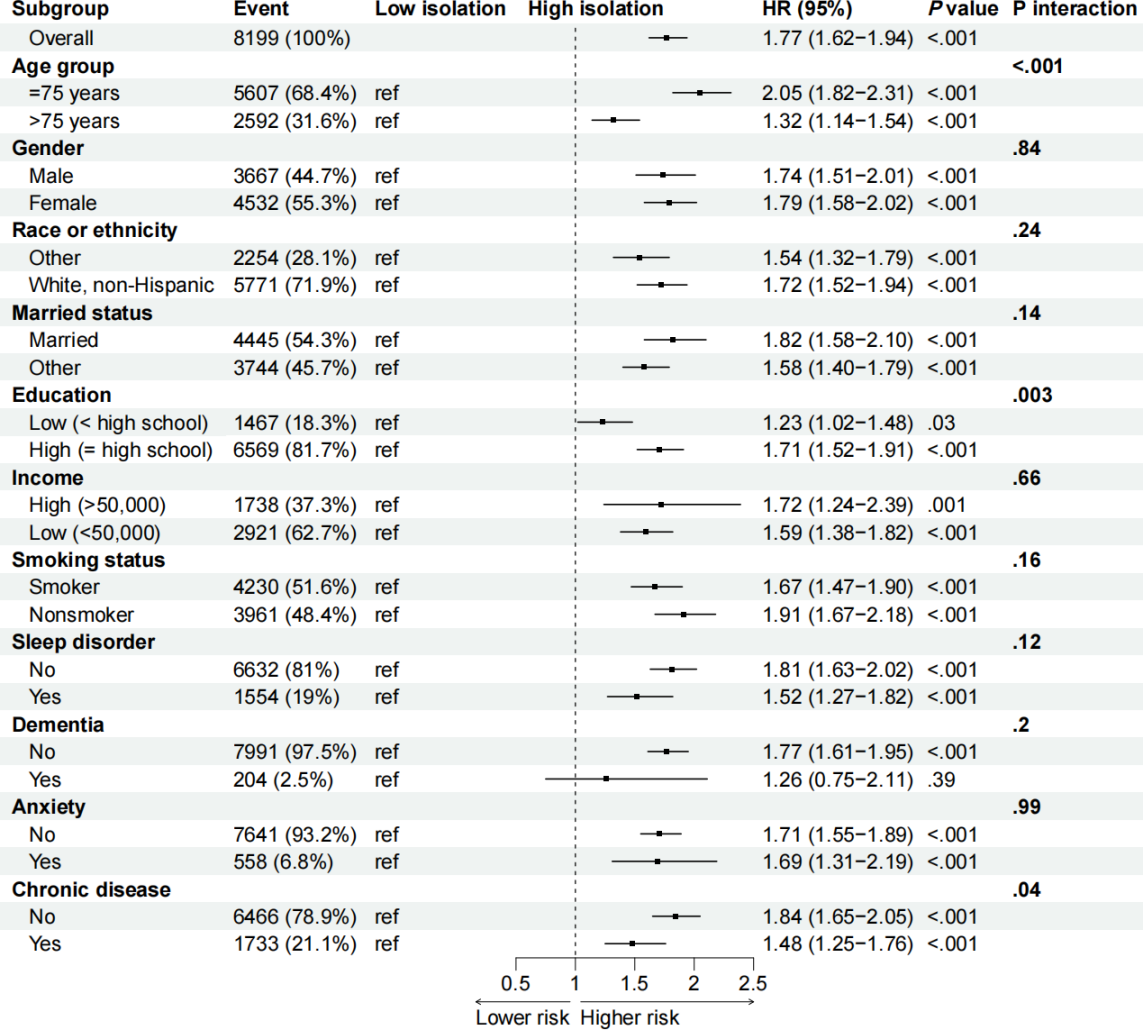
The relationship between digital isolation and depression across subgroups.

Table 2 demonstrates a significant correlation between heightened digital isolation and a greater likelihood of depression. The Cox proportional hazards analysis indicated that participants in the high-isolation category had a notably higher risk of depression compared to those in the low-isolation category. In the discovery cohort, the unadjusted HR for the high-isolation group was 1.73 (95% CI 1.54‐1.94; *P* <.001), which reduced to an adjusted HR of 1.33 (95% CI 1.12‐1.57; *P*=.001) upon controlling for potential confounders. The validation cohort displayed comparable results, with an unadjusted HR of 1.81 (95% CI 1.55‐2.12; *P*<.001) and an adjusted HR of 1.37 (95% CI 1.07‐1.75; *P*=.012). When analyzing the combined sample, the unadjusted HR was 1.75 (95% CI 1.60‐1.92; *P*<.001), and the adjusted HR was 1.35 (95% CI 1.18‐1.55; *P*<.001). Figure 2 visually supports these findings, depicting a significantly higher cumulative incidence of depression in the high-isolation group throughout the follow-up period. Figure 3 presents the stratified analyses examining the association between digital isolation and depression across various subgroups. Formal tests for interaction revealed that the effect of digital isolation on depression risk was significantly modified by age, education level, and chronic disease burden. Specifically, the association was stronger in individuals aged 75 years or younger compared to those older than 75 years (*P* for interaction <.001), in those with higher education (*P* for interaction=.003), and in those with fewer than 3 chronic diseases (*P* for interaction=.04). No significant interaction effects were observed for other subgroups, including gender, race or ethnicity, and marital status, suggesting the association was broadly consistent across these groups. Collectively, the data from Table 2 and Figures2  3 strongly suggest that increased digital isolation is significantly linked to a higher risk of depression, a relationship that persists even after accounting for multiple potential confounding factors. This underscores the necessity of incorporating digital isolation reduction strategies into public health initiatives to mitigate the burden of depression.

**Table 2.** The observed link between digital isolation and the likelihood of depression.

Samples	Event and total number (%)	Model 1 HR (95% CI)^a^^,^^b^	*P* value	Model 2 HR (95% CI)^b^^,^^c^	*P* value
Discovery					
Low isolation (reference)	3408/5169 (65.93)	1 (reference)		1 (reference)	
High isolation	1761/5169 (34.07)	1.73 (1.54-1.94)	<.001	1.33 (1.12-1.57)	.001
Validation					
Low isolation (reference)	2250/3030 (74.26)	1 (reference)		1 (reference)	
High isolation	780/3030 (25.74)	1.81 (1.55-2.12)	<.001	1.37 (1.07-1.75)	.01
Pooled					
Low isolation (reference)	5658/8199 (69.01)	1 (reference)		1 (reference)	
High isolation	2541/8199 (30.99)	1.75 (1.60-1.92)	<.001	1.35 (1.18-1.55)	<.001

a Model 1: unadjusted Cox proportional hazards model.

b HR: hazard ratio.

c Model 2: Cox proportional hazards model adjusted for age, gender, race, education, income, marital status, sleep disorder, smoking status, dementia, anxiety, and chronic disease.

### Association Between Different Items of Digital Isolation and Depression

An overview of the correlation between the various components of digital isolation and the likelihood of depression is provided in Table 3. Adjusted Cox models revealed significant associations for computer isolation, email isolation, and online isolation, but not for phone isolation. In the pooled sample, adjusted HRs were 1.23 (95% CI 1.08‐1.41; *P*=.003) for computer isolation, 1.52 (95% CI 1.32‐1.75; *P*<.001) for email isolation, and 1.32 (95% CI 1.15‐1.52; *P*<.001) for online isolation. In contrast, the adjusted HR for phone isolation was 1.19 (95% CI 0.98‐1.44; *P*=.08), which was not statistically significant. These findings suggest that isolation related to more complex digital activities, such as computer, email, and internet use, has a stronger and more significant association with depression risk in comparison to the weaker and nonsignificant association observed for mobile phone isolation.

**Table 3.** Association between different items of digital isolation and depression.

Sample or variables	Model 1 HR^a^ (95% CI)	*P* value	Model 2 HR (95% CI)	*P* value
Discovery sample				
Phone isolation	1.29 (1.09-1.54)	.004	1.18 (0.94-1.47)	.15
Computer isolation	1.55 (1.38-1.75)	<.001	1.19 (1.01-1.40)	.04
Email isolation	1.81 (1.60-2.05)	<.001	1.48 (1.24-1.77)	<.001
Online isolation	1.76 (1.56-1.99)	<.001	1.35 (1.13-1.61)	.001
Validation sample				
Phone isolation	1.34 (1.00-1.79)	.05	1.24 (0.85-1.83)	.27
Computer isolation	1.74 (1.49-2.02)	<.001	1.28 (1.02-1.61)	.03
Email isolation	1.99 (1.71-2.31)	<.001	1.59 (1.25-2.02)	<.001
Online isolation	1.86 (1.60-2.17)	<.001	1.26 (0.99-1.60)	.06
Pooled sample				
Phone isolation	1.30 (1.12-1.51)	<.001	1.19 (0.98-1.44)	.08
Computer isolation	1.62 (1.47-1.78)	<.001	1.23 (1.08-1.41)	.003
Email isolation	1.86 (1.69-2.04)	<.001	1.52 (1.32-1.75)	<.001
Online isolation	1.79 (1.63-1.97)	<.001	1.32 (1.15-1.52)	<.001

a HR: hazard ratio.

### Nonlinear Relationship Between Digital Isolation and Depression Risk

Multimedia Appendix 2 presents results from RCS analyses examining nonlinear relationships between digital isolation scores (0‐4) and depression risk. Across all cohorts—discovery, validation, and pooled—a consistent nonlinear trend was observed, with depression risk increasing progressively with higher digital isolation scores. This trend reinforces the role of digital isolation as a significant contributor to depression risk.

### Sensitivity Analysis

Sensitivity analyses confirmed the robustness of the main findings. Using a multilevel categorization of digital isolation (no isolation, moderate isolation, and high isolation; Table S1 in MultimediaAppendices 1  3), the results were consistent across discovery, validation, and pooled samples. To address potential concerns regarding the combination of 2 cohorts, interaction analyses between digital isolation and cohort membership were conducted. As shown in Table S2 (Multimedia Appendix 1), no significant interaction was found in either unadjusted or adjusted models (all *P*>.05), confirming the consistency of digital isolation effects across both cohorts. The adjusted HRs in the combined analysis revealed a significant association between isolation and adverse outcomes. Specifically, moderate isolation was associated with an HR of 1.63 (95% CI 1.44‐1.84; *P*<.001), whereas high isolation carried an even higher HR of 2.23 (95% CI 1.99‐2.51; *P*<.001), both in comparison to the absence of isolation. KM survival curves further supported these findings, showing significantly higher depression incidence over time in the high isolation group. The sensitivity analyses emphasized the strong and consistent link between digital isolation and depression risk, thereby underscoring the reliability of the findings across different analytical methods and stratifications. After further adjustment for in-person social isolation, the association between digital isolation and depression remained robust across the discovery, validation, and pooled cohorts (Table S3 in Multimedia Appendix 1). Although the HRs were slightly attenuated in Model 3, the associations remained statistically significant, supporting the independent contribution of digital isolation to depression risk.

## Discussion

### Principal Results

The study demonstrates a strong association between digital isolation and the increased likelihood of developing depression. Using Cox proportional hazards models, researchers observed that individuals in the high-isolation category exhibited a significantly elevated risk of depression compared to those in the low-isolation group, specifically in terms of computer isolation, email isolation, and online isolation. Additionally, the analysis using KM survival curves confirmed these findings, revealing a notably higher cumulative incidence of depression over time within the high-isolation group. These outcomes emphasize digital isolation as a crucial risk factor for depression.

This study’s results support existing research that demonstrates a connection between digital isolation and negative mental health consequences. Notably, prior investigations have consistently found that social isolation and restricted access to digital resources are strongly linked to increased rates of depression [14,27,28]. Our study builds on these findings, highlighting the pronounced effects of computer, email, and online isolation. Notably, in contrast to some earlier research, we observed that the impact of mobile phone isolation was relatively limited. This may be due to the high prevalence of mobile phone adoption among older adults. As shown in Table 1, only 8.6% of the pooled sample experienced phone isolation, compared to 30.0% for computer isolation and more than 50% for email and online isolation. Because the vast majority of older adults in this cohort use mobile phones, the small group that does not may be too heterogeneous to show a consistent effect. The relationship between digital isolation and depression may operate through several psychosocial mechanisms. First, digital isolation can foster social isolation by reducing interpersonal interactions and social support, thereby increasing vulnerability to depression. Second, restricted access to online information may limit awareness and use of mental health resources, exacerbating depressive symptoms. In particular, computer and online isolation may more severely restrict access to information and social networks, potentially explaining their stronger associations with depression observed in this study. Notably, our subgroup analyses revealed a more complex relationship than anticipated. The detrimental effect of digital isolation was paradoxically stronger in individuals who were younger (≤75 years), had higher education, and were healthier (fewer than 3 chronic diseases). These unexpected findings challenge a simplistic view of vulnerability, suggesting that the psychological reliance on digital connectivity—and the subsequent impact of being disconnected—may differ substantially across demographic groups. The mechanisms driving these specific interactions represent a critical area for future investigation.

### Strengths

One of the key strengths of our research lies in the adoption of a longitudinal cohort design. This approach permitted us to monitor participants’ progress over an extended period, thereby enhancing our ability to establish a clearer causal link between digital isolation and depression. Additionally, we used a large sample size and performed dual analysis using discovery and validation cohorts to ensure the robustness and reproducibility of our results. This design enhances the external validity of the study, augmenting the generalizability of the findings. We also adjusted for a variety of potential confounding factors, such as demographic characteristics, health status, and lifestyle factors, further enhancing the accuracy of the analysis. Further analysis revealed a nonlinear association between digital isolation scores and depression risk. The RCS analysis (Multimedia Appendix 2) showed a nonlinear upward trend in depression risk as digital isolation scores increased, underscoring the complexity of how digital isolation affects mental health. These results support the conclusion that higher digital isolation levels substantially elevate depression risk. Sensitivity analyses (Table S1 in MultimediaAppendices 1  3) confirmed the robustness of these findings, demonstrating consistent results across different grouping strategies. These additional analyses further strengthen the link between digital isolation and depression risk, thereby enhancing the credibility and reliability of the study’s conclusions.

### Limitations

Despite its strengths, including a large, longitudinal, and nationally representative sample, this study has several limitations. First, our measures have inherent constraints. The digital isolation index, while based on prior research, has not been formally validated, and its binary (use vs. nonuse) operationalization does not capture the nuances of engagement frequency or quality. Furthermore, both digital use and depressive symptoms were self-reported, which may be subject to recall bias. Besides, the use of a 1-month recall period for the PHQ-2, a deviation from the standard 2-week timeframe for which the instrument was validated, may affect the comparability of our findings and could potentially influence symptom reporting. Second, challenges to causal inference remain. Despite adjusting for in-person social isolation, the potential for unmeasured confounding from factors such as mobility limitations, cognitive decline, or health care accessibility persists. Additionally, while our longitudinal design mitigates the risk, we cannot fully rule out reverse causality, where subclinical depressive symptoms might precede digital withdrawal. Future research using advanced methods such as cross-lagged panel models is needed to further disentangle this relationship. Finally, the generalizability of our findings may be limited. The results are based on community-dwelling older adults in the United States and may not apply to institutionalized individuals, younger populations, or those in different cultural and technological contexts. This nonuse-based proxy primarily captures the quantity and frequency of participation, but it does not directly assess the quality of interactions or subjective experiences of digital loneliness. Additionally, some instances of nonuse may be voluntary.

### Future Directions and Public Health Relevance

Our findings have direct public health implications, indicating that promoting digital literacy among older adults is essential for safeguarding their mental health through targeted policies and interventions. To strengthen this observational evidence, future studies should prioritize establishing causality through experimental designs, such as randomized controlled trials of digital literacy interventions. Methodologically, advanced techniques such as cross-lagged panel models are needed to further address the possibility of reverse causality. Finally, the research scope should be expanded to explore the effects of digital isolation on additional outcomes, including anxiety and cognitive decline, as well as to examine the complex role of emerging digital platforms.

### Conclusions

The study reveals a strong connection between digital isolation and the likelihood of depression, with specific emphasis on computer isolation, email detachment, and internet seclusion. Though mobile phone isolation seems to have a lesser effect, the overall outcomes emphasize the vital need to combat digital isolation in order to foster improved mental health. These findings hold substantial relevance for the development of public health policies and interventions, particularly targeting older individuals and other vulnerable groups, to alleviate digital isolation and its associated risks.

## Supplementary material

10.2196/75174 Multimedia Appendix 1 Additional analyses, including sensitivity analyses, interaction effects, item-level associations of digital isolation, and detailed construction of the digital isolation index used in the study.

10.2196/75174 Multimedia Appendix 2 Nonlinear association between digital isolation score and depression risk in discovery, validation, and pooled samples (restricted cubic splines analysis).

10.2196/75174 Multimedia Appendix 3 Kaplan-Meier survival curves showing depression risk by digital isolation levels (sensitivity analysis).

## Abbreviations

HR: hazard ratio
KM: Kaplan-Meier
NHATS: National Health and Aging Trends Study
PHQ-2: Patient Health Questionnaire-2
RCS: restricted cubic splines
