# Supplementary material for: Digital Isolation and Depression Risk in Older Adults Using the National Health and Aging Trends Study Database: 8-Year Longitudinal Study
Source: JMIR Aging. 2025 Dec 12;8:e75174. doi: 10.2196/75174 (PMC12700337; doi:10.2196/75174)
Supplement: Multimedia Appendix 1 [file aging-v8-e75174-s001.docx]

**Table S1. Association between digital isolation and the risk of depression (sensitivity analysis).**

| **Sample/Variables** | **Event/N (%)** | **Model 1 HR (95% CI)^a,c^** | **P-Value** | **Model 2 HR (95% CI)^b,c^** | **P-Value** |
| --- | --- | --- | --- | --- | --- |
| **Discovery Sample** |  |  |  |  |  |
| No Isolation (Ref) | 707/3118 (22.67) | 1 (Ref) |  | 1 (Ref) |  |
| Moderate Isolation | 242/1474 (16.42) | 1.54 (1.33, 1.78) | <0.001 | 1.56 (1.33, 1.83) | <0.001 |
| High Isolation | 226/577 (39.17) | 2.12 (1.88, 2.54) | <0.001 | 2.20 (1.89, 2.56) | <0.001 |
| **Validation Sample** |  |  |  |  |  |
| No Isolation (Ref) | 423/1753 (24.13) | 1 (Ref) |  | 1 (Ref) |  |
| Moderate Isolation | 169/1173 (14.41) | 1.76 (1.46, 2.14) | <0.001 | 1.76 (1.46, 2.13) | <0.001 |
| High Isolation | 92/104 (88.46) | 2.31 (1.91, 2.80) | <0.001 | 2.31 (1.91, 2.80) | <0.001 |
| **Pooled Sample** |  |  |  |  |  |
| No Isolation (Ref) | 1130/4871 (23.20) | 1 (Ref) |  | 1 (Ref) |  |
| Moderate Isolation | 411/2647 (15.53) | 1.63 (1.44, 1.84) | <0.001 | 1.63 (1.44, 1.84) | <0.001 |
| High Isolation | 318/681 (46.69) | 2.23 (1.99, 2.51) | <0.001 | 2.23 (1.99, 2.51) | <0.001 |

**Table S2. Interaction between digital Isolation and cohort**

| Model | Interaction Term (Digital Isolation × Cohort^c^) | P-value |
| --- | --- | --- |
| Model 1^a^ | HR = 1.04 (95% CI: 0.86–1.27) | 0.657 |
| Model 2^b^ | HR = 1.13 (95% CI: 0.87–1.46) | 0.354 |

**^a^Model 1**: Unadjusted Cox proportional hazards model.

**^b^Model 2**: Cox proportional hazards model adjusted for age, gender, race, baseline disease, depression, anxiety, smoking status, and sleep difficulty.

All variables compare "Not using" vs. "Using."

^c^Cohort including discovery and validation cohorts

**Table S3. Association between different items of digital isolation and depression (adjusted for in-person social isolation)**

| **Samples** | **Event/Total number (%)** | **Model 1 HR (95% CI)^a,d^** | **P-Value** | **Model 2 HR (95% CI)^b,d^** | **P-Value** | **Model 3 HR (95% CI)^c,d^** | **P-Value** |
| --- | --- | --- | --- | --- | --- | --- | --- |
| **Discovery** |  |  |  |  |  |  |  |
| Low Isolation (Ref) | 3,408/5,169 (65.93) | 1 (Ref) |  | 1 (Ref) |  | 1 (Ref) |  |
| High Isolation | 1,761/5,169 (34.07) | 1.73 (1.54, 1.94) | <0.001 | 1.33 (1.12, 1.57) | 0.001 | 1.28 (1.08, 1.52) | 0.004 |
| **Validation** |  |  |  |  |  |  |  |
| Low Isolation (Ref) | 2,250/3,030 (74.26) | 1 (Ref) |  | 1 (Ref) |  | 1 (Ref) |  |
| High Isolation | 780/3,030 (25.74) | 1.81 (1.55, 2.12) | <0.001 | 1.37 (1.07, 1.75) | 0.012 | 1.32 (1.02, 1.70) | 0.033 |
| **Pooled** |  |  |  |  |  |  |  |
| Low Isolation (Ref) | 5,658/8,199 (69.01) | 1 (Ref) |  | 1 (Ref) |  | 1 (Ref) |  |
| High Isolation | 2,541/8,199 (30.99) | 1.75 (1.60, 1.92) | <0.001 | 1.35 (1.18, 1.55) | <0.001 | 1.30 (1.14, 1.49) | <0.001 |

**^a^Model 1:** Unadjusted Cox proportional hazards model.

**^b^Model 2:** Cox proportional hazards model adjusted for age, gender, race, education, income, marital status, sleep disorder, smoking status, dementia, anxiety, and chronic disease.

**^C^Model 3: Model 2+in-person social isolation**

^d^**Abbreviation**: HR, Hazard Ratio; CI, Confidence Interval.

**Table S4. Construction of the digital isolation index from NHATS round 1 questionnaire items.**

| **Component of Digital Isolation** | **NHATS Variable(s)** | **Question Text (Abbreviated)** | **Coding for Isolation (Score = 1)** |
| --- | --- | --- | --- |
| Phone Isolation | te1cellphone | "Do you have a working cell phone?" | Response is 'NO' (Code 2) |
| Computer Isolation | te1computer & te1compoth | "Do you have a working computer at home?" & "In the last month, have you used a computer anywhere else?" | Response is 'NO' to both questions (TE6=2 AND TE8=2) |
| Electronic Communication Isolation | te1emailtext | "In the last month, have you ever sent messages by email or texting?" | Response is 'NO' (Code 2) |
| Internet Activity Isolation | te1online | "In the last month, have you ever gone on the Internet or online for any other reason?" | Response is 'NO' (Code 2) |
