# Supplementary material for: Digital Isolation and Depression Risk in Older Adults Using the National Health and Aging Trends Study Database: 8-Year Longitudinal Study
Source: JMIR Aging. 2025 Dec 12;8:e75174. doi: 10.2196/75174 (PMC12700337; doi:10.2196/75174)
Supplement: Multimedia Appendix 2 [file aging-v8-e75174-s002.docx]

**Figure S1:** Non-linear association between digital isolation score and depression risk in discovery, validation, and pooled samples (restricted cubic splines analysis).


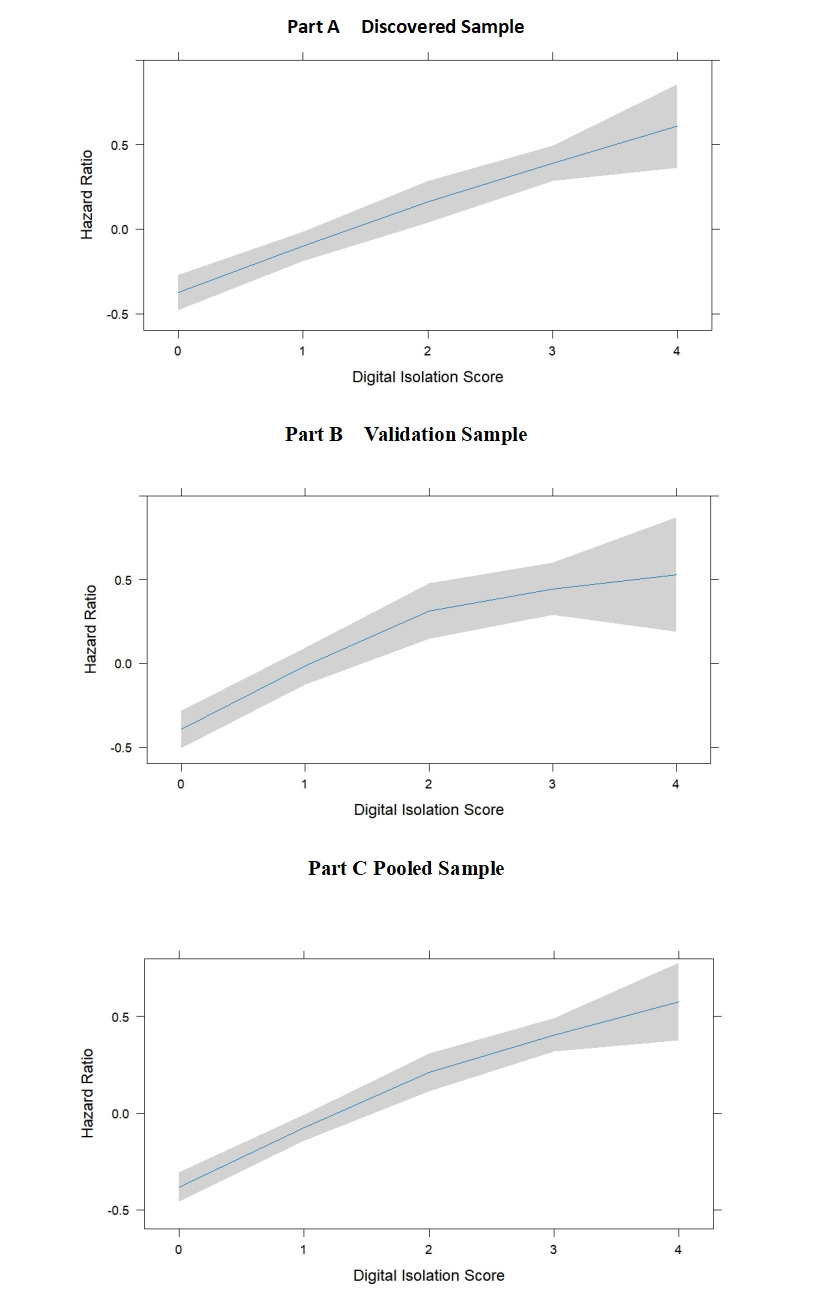


This figure depicts the non-linear relationship between digital isolation scores (0–4) and depression risk, analyzed using RCS across three cohorts. HRs are plotted on the y-axis, with the reference value set at 0 (no isolation). Shaded areas indicate 95% confidence intervals.

**Part A**: Discovery sample (n = 5,169), showing a steady increase in depression risk with higher digital isolation scores.

**Part B:** Validation sample (n = 3,030), revealing similar trends, albeit with wider confidence intervals due to the smaller sample size.

**Part C:** Pooled sample (n = 8,199), integrating discovery and validation data to provide robust estimates.

The analysis reveals a dose-response relationship, with depression risk progressively rising as digital isolation scores increase. These results support the hypothesis that digital isolation is a significant and incremental risk factor for depression. The RCS method enables flexible modeling of non-linear effects, offering detailed insights into the complex interplay between digital isolation and mental health outcomes.
