# Supplementary material for: Digital Isolation and Depression Risk in Older Adults Using the National Health and Aging Trends Study Database: 8-Year Longitudinal Study
Source: JMIR Aging. 2025 Dec 12;8:e75174. doi: 10.2196/75174 (PMC12700337; doi:10.2196/75174)
Supplement: Multimedia Appendix 3 [file aging-v8-e75174-s003.docx]

**Figure S2:** Kaplan-Meier survival curves showing depression risk by digital isolation levels (sensitivity analysis).


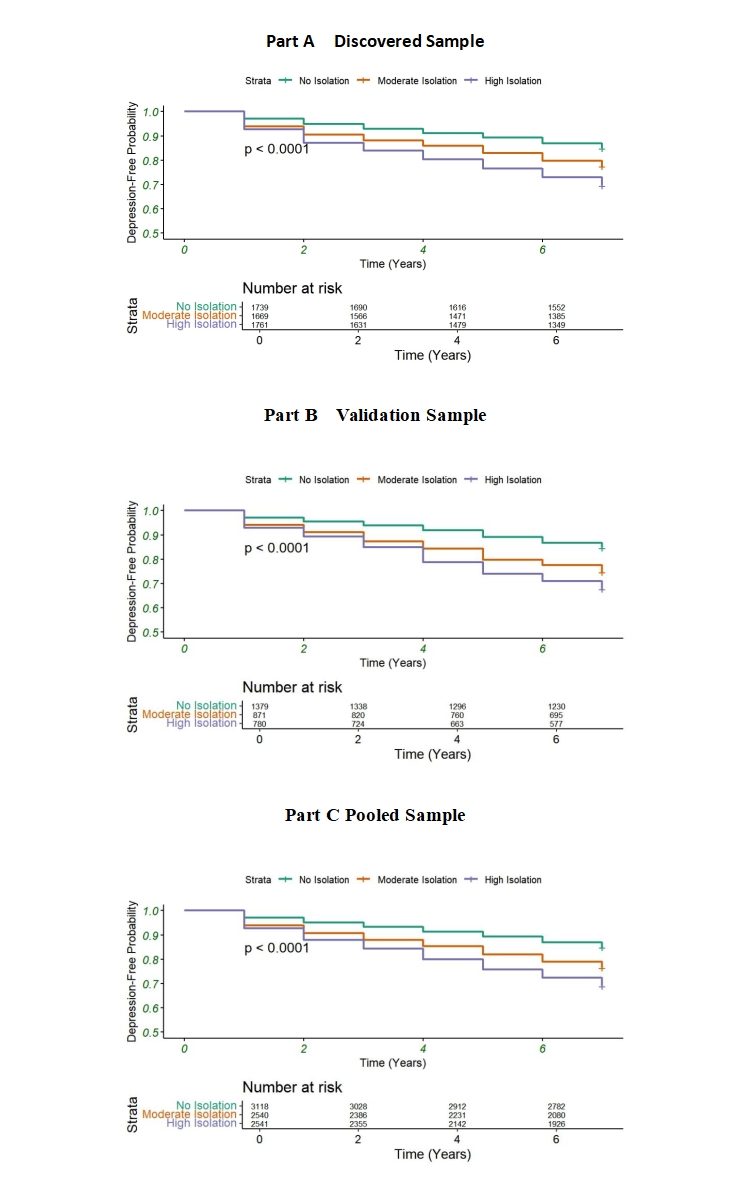


This figure illustrates Kaplan-Meier survival curves for depression-free probability, stratified by digital isolation levels in the sensitivity analysis. Digital isolation was categorized into three levels: No Isolation (0 points), Moderate Isolation (1–2 points), and High Isolation (3–4 points). Survival probabilities for each group are plotted over time (years).

**Part A:** Discovery sample (n = 5,169).

**Part B:** Validation sample (n = 3,030).

**Part C:** Pooled sample (n = 8,199).

P-values from log-rank tests comparing survival distributions across the three groups are shown in each panel. The number of participants at risk at each time point is provided below the plots. The results consistently demonstrate a significant association between higher digital isolation levels and increased depression risk in all samples.
